# *Poria Cocos* Ameliorates Bone Loss in Ovariectomized Mice and Inhibits Osteoclastogenesis In Vitro

**DOI:** 10.3390/nu12051383

**Published:** 2020-05-12

**Authors:** Youn-Hwan Hwang, Seon-A Jang, Ami Lee, Taesoo Kim, Hyunil Ha

**Affiliations:** 1 Herbal Medicine Research Division, Korea Institute of Oriental Medicine, Yuseong-daero 1672, Yuseong-gu, Daejeon 34054, Korea; hyhhwang@kiom.re.kr (Y.-H.H.); white7068@kiom.re.kr (S.-A.J.); dmb01367@kiom.re.kr (A.L.); xotn91@kiom.re.kr (T.K.); 2 University of Science & Technology (UST), Korean Convergence Medicine Major KIOM, 1672 Yuseongdae-ro, Yuseong-gu, Daejeon 34054, Korea

**Keywords:** *Poria cocos*, osteoporosis, ovariectomy, osteoclast, menopause

## Abstract

Estrogen deprivation in postmenopausal women causes disruption of bone homeostasis, resulting in bone loss and osteoporosis. Conventional therapies can exert adverse effects. The sclerotum of *Poria cocos* has been used in traditional medicine and as a nutritional supplement and to treat various diseases. However, the effects of *P. cocos* on the bone remain largely undetermined. In this study, we examined the effects of *P. cocos* hydroethanolic extract (PC) on osteoclast differentiation and estrogen-deprivation-induced bone loss in an ovariectomized mouse model of postmenopausal osteoporosis. PC-mediated inhibition of receptor activator of nuclear factor-κB ligand (RANKL)-induced osteoclast formation and resorption activity suppressed RANKL-induced expression of nuclear factor of activated T cells cytoplasmic 1 (NFATc1), which is a crucial transcription factor for osteoclast differentiation. In ovariectomized mice, PC markedly alleviated trabecular bone loss and reduced the accumulation of lipid droplets in the bone marrow. We additionally identified ten triterpenoid constituents of PC using UPLC-MS/MS analysis. Our results indicate that PC negatively regulated osteoclast differentiation and function, and can potentially be used to manage postmenopausal osteoporosis.

## 1. Introduction

Bone is a highly dynamic organ, whose structure maintenance involves continual remodeling by bone-resorbing osteoclasts and bone-forming osteoblasts [1]. Bone remodeling is commonly deteriorative in osteolytic disorders such as osteoporosis, osteoarthritis, and bone metastases. Estrogen deprivation in postmenopausal women causes disruption of bone homeostasis, resulting in bone loss and osteoporosis [2]. Conventional therapies for osteoporosis, such as hormone-replacement therapy, bisphosphonates, and selective estrogen receptor modulators, are effective, but can exert adverse effects and do not meet all the needs of these patients [3]. Natural products and traditional medicines used to manage osteoporosis have been garnering increased attention because they are suitable for long-term use [4]. Recently, conventional therapy combined with nutraceutical food supplementation, such as herbal derivatives, isoflavones, polyphenols, and vitamins, has been reported to have beneficial effects in experimental studies and clinical trials [5,6].

*Poria cocos* Wolf, a fungus in the family Polyporaceae, is known as “Bokryung” in Korea, “Fu Ling” in China, and “Indian bread” in North America [7]. In East Asian and European countries, the sclerotum of *P. cocos* has commonly been used therapeutically against various diseases, and as a functional food and dietary ingredient in tea, bread, and other baked goods [8]. *P. cocos* exerts beneficial effects on patients with insomnia, heart disorders, chronic edema, and nephrosis [9]. Recent pharmacological studies show that *P. cocos* and its constituents including polysaccharides possesses immunomodulatory, anti-inflammatory, anti-cancer, anti-hyperglycemic, and anti-nephritic activity [10,11]. Two previous studies have shown that three diterpenes and polysaccharides isolated from *P. cocos* positively modulate osteoblastic differentiation [7] and osteoclast formation [12], respectively, in vitro. However, the anti-osteoporotic properties of *P. cocos* remain undetermined. In this study, we investigated the inhibitory effects of hydroethanolic extract of *P. cocos* (PC) on osteoclast differentiation and function, and evaluated the beneficial effects of PC on ovariectomy (OVX)-induced bone loss in mice.

## 2. Materials and Methods

### 2.1. Materials

The α-minimal essential medium (α-MEM), fetal bovine serum (FBS), acetonitrile, water, formic acid, high-capacity cDNA reverse transcription kit, and bicinchoninic acid assay (BCA) kit were purchased from Thermo Fisher Scientific (Waltham, MA, USA). TaqMan primers for c-Fos (Mm00487425_m1), nuclear factor of activated T-cells and cytoplasmic 1 (NFATc1, Mm00479445_m1), Atp6v0d2 (Mm00656638_m1), cathepsin K (Mm00484036_m1), dendritic cell-specific transmembrane proteins (Dcstamp, Mm01168058_m1), 18S rRNA (Hs99999901_s1), and universal PCR master mix were purchased from Applied Biosystems (Foster City, CA, USA). The RNeasy kit was obtained from Qiagen (Hilden, Germany). NFATc1, c-Fos, β-actin, and secondary antibodies were purchased from Santa Cruz Biotechnology (Santa Cruz, CA, USA), and the p38 antibody was obtained from Cell Signaling Technology (Danvers, MA, USA). PC was purchased from the National Development Institute of Korean Medicine (Gyeongsan, Republic of Korea) and stored in the herbarium (voucher number #KE-1) of the Herbal Medicine Research Division. Dried PC (0.5 kg) was extracted using 70% ethanol in distilled water (3.5 L, *v*/*v*) under reflux for 3 h, and lyophilized after filtration. PC powder was stored at −20 °C until further use.

### 2.2. Animals

Female C57BL/6 mice (6 weeks old) were purchased from Japan SLC (Shizuoka, Japan) and acclimated for 7 days under standard housing conditions. Mice were fed a standard-chow diet (Cargil, Pyengtaek, Republic of Korea) before being switched to a high-fat diet (HFD; 60 kcal%; Research Diet, New Brunswick, NJ, USA) on day 7 post-surgery. Water and feed were provided to the mice ad libitum. All procedures involving animals were approved by the Institutional Animal Care and Use Committee of KNOTUS Co., Ltd. (Guri, Republic of Korea).

### 2.3. Osteoclast Differentiation and Resorption Pit Assay

Bone-marrow-derived macrophages (BMMs) were cultured in α-MEM, containing 10% FBS and macrophage colony-stimulating factor (M-CSF, 30 ng/mL), as described previously [13]. BMMs (1 × 10^4^ cells/well in a 96-well plate or 1 × 10^5^ cells/well in a 6-well plate) were treated with M-CSF and RANKL (100 ng/mL) for 4 days to induce osteoclast differentiation with or without PC. Tartrate-resistant acid phosphatase (TRAP) activity, which is a marker for osteoclast differentiation, was examined by TRAP staining as described previously [10]. For resorption pit assay, BMMs were seeded at 1.5 × 10^4^ cells/well in hydroxyapatite-coated plates (Corning Inc., NY, USA) and treated with M-CSF and RANKL, with or without PC, for 4 days. The relative area of resorption pits, after cells were removed by using hypochlorite solution, was measured using ImageJ software (National Institutes of Health, Bethesda, MD, USA).

### 2.4. Animal Study and Micro-computed Tomography (μ-CT)

All procedures involving animals and μ-CT analysis were performed in accordance with previously described protocols [14]. Briefly, mice were sham- (normal control; *n* = 8), or OVX-operated using bilateral dorsal incision under Zoletil 50 (Virbac, Carros, France) and Rumpun (Bayer, Leverkusen, Germany)-induced anesthesia. OVX mice were randomly divided into three groups (*n* = 8 mice per group) and treated with vehicle (used as negative control), low-dose PC (PC L; 10 mg/kg), or high-dose PC (PC H; 20 mg/kg). On day 7 post-surgery, PC was administered to the mice by oral gavage once daily for 4 weeks. Gonadal fat and femurs were collected from the mice after they were humanely euthanized using Zoletil and Rumpun. The femurs were fixed in 10% phosphate-buffered formalin, decalcified in RDO Gold (RDO, Aurora, IL, USA), then embedded in paraffin, dewaxed, and stained with hematoxylin and eosin.

The microarchitecture of distal femurs was measured and analyzed using a Quantume GX μCT imaging system (PerkinElmer, Hopkinton, MA, USA). After reconstruction of the scanned images, trabecular bone parameters were calculated using SkyScan software (Bruker, Kontich, Belgium). Bone volume to tissue volume ratio (BV/TV), trabecular number (Tb.N), trabecular separation (Tb.S), and trabecular thickness (Tb.Th) were then determined.

### 2.5. Real-time Quantitative PCR and Western Blotting

Total RNA was extracted using the RNeasy kit, and cDNA was reverse-transcribed using a High-Capacity cDNA Reverse Transcription Kit. Real-time PCR was performed using an ABI 7500 Real-Time PCR system (Applied Biosystems, Waltham, MA, USA) with TaqMan primers for c-Fos, NFATc1, Atp6v0d2, Ctsk, and Dcstamp, and Universal PCR Master Mix, according to the manufacturers’ instructions. Target mRNA levels were calculated and normalized to those of 18S rRNA.

Whole cell lysates were extracted using lysis buffer containing protease and phosphatase inhibitors in accordance with a method reported previously [15]. Protein content was quantitated using a BCA kit. Equal amounts of protein were resolved using sodium dodecyl sulfate (SDS)–polyacrylamide gel electrophoresis and transferred to a polyvinylidene difluoride (PVDF) membrane. The membrane was incubated for 1 h with primary antibodies (1:1000 dilution) against c-Fos, NFATc1, and p38, and then with secondary antibodies (1:2000 dilution). Signal intensity was detected using the ChemiDoc imaging system (Bio-Rad, Hercules, CA, USA) with chemiluminescence reagent.

### 2.6. Ultrahigh-performance Liquid Chromatography-diode Array Detector–tandem Mass Spectrometry (UHPLC-DAD–MS/MS) analysis

The phytochemical constituents in PC were identified using a Dionex UltiMate 3000 system coupled with a Thermo Q-Exactive mass spectrometer as described previously with some modifications [16]. An Acquity BEH C18 column (150 × 2.1 mm, 1.7 μm) with 0.1% formic acid in water and acetonitrile was used, and gradient elution was performed. A Q-Exactive mass spectrometer was equipped with an electrospray ionization source and operated in negative ion mode. Spectral data were obtained in full MS1 and data-dependent MS2 scan mode. The full acquisition parameters were as follows: resolution, 70,000; scan range, 100–1500 m/z. MS2 parameters were as follows: resolution, 17,500; normalized collision energy, 40. Data acquisition and analysis were performed using Xcalibur software (Thermo Fisher Scientific, Foster City, CA, USA).

### 2.7. Statistical Analysis

Data are presented as mean ± standard error of the mean (SEM) for the in vivo study, and mean ± standard deviation (SD) for the in vitro study. Data were analyzed via one-way analysis of variance (ANOVA) with Dunnett’s post hoc test using Prism (Graphpad, San Diego, CA, USA). *p*-values less than 0.05 were considered significant.

## 3. Results and Discussion

### 3.1. PC inhibits Osteoclastogenesis and Resorption Activity

**Figure 1:**
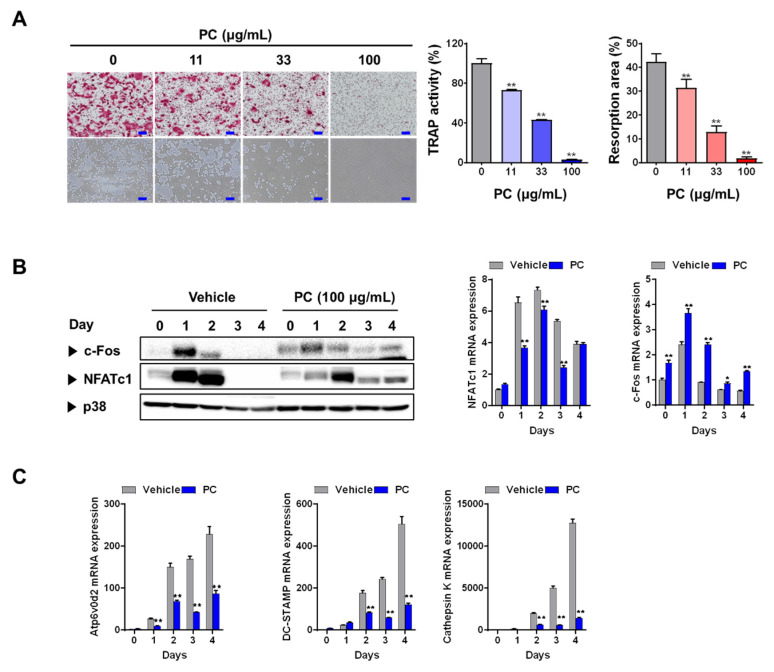
*P. cocos* hydroethanolic extract (PC) inhibits RANKL-induced osteoclastogenesis in bone-marrow-derived macrophages (BMMs). (**A**) Inhibitory effects of PC on osteoclast differentiation. BMMs treated with vehicle (distilled water) or PC were evaluated using tartrate-resistant acid phosphatase (TRAP) staining for TRAP activity, and using resorption pits and area. (**B**) PC-mediated effects on the expression of c-Fos and NFATc1. (**C**) PC modulated the expression of osteoclast-specific genes. For Western blotting and real-time qPCR, BMMs were pretreated with or without PC for 3 h, and then treated with RANKL (100 ng/mL) for the indicated number of days. Data are represented as mean ± SD of three independent experiments. * *p* < 0.05, ** *p* < 0.01 vs. control treated with vehicle.

Osteoclasts, which are specialized bone-resorbing cells, are derived by proliferation, differentiation, and fusion of a monocyte/macrophage lineage under the regulation of various genetic, humoral, and mechanical factors during a multi-stage process. This process, called osteoclastogenesis, is mainly regulated by the key cytokines M-CSF and RANKL [17]. RANKL and M-CSF can differentiate BMM osteoclast precursors into osteoclasts. The fully differentiated osteoclasts attach to bone surface and digest the bone organic matrix via secretion of acids and proteases such as cathepsin K [18,19]. To characterize the effects of PC on RANKL-induced osteoclast differentiation, we used different PC concentrations (0–100 μg/mL) to treated BMMs that had also been pretreated with M-CSF (30 ng/mL) and RANKL (100 ng/mL). Our results show that PC markedly reduced not only the formation of TRAP-positive multinucleated osteoclasts, but also their TRAP activity, in a dose-dependent manner compared with those of vehicle-treated controls (*p* < 0.01, Figure 1A). In agreement with our results on osteoclast formation, PC suppressed osteoclast resorption activity on hydroxyapatite matrix (*p* < 0.01). Differentiated osteoclasts exhibit high TRAP activity, which is a biomarker for osteoclast maturation; therefore, these findings indicate that PC inhibited osteoclast differentiation and bone-resorbing activity.

**Figure 2:**
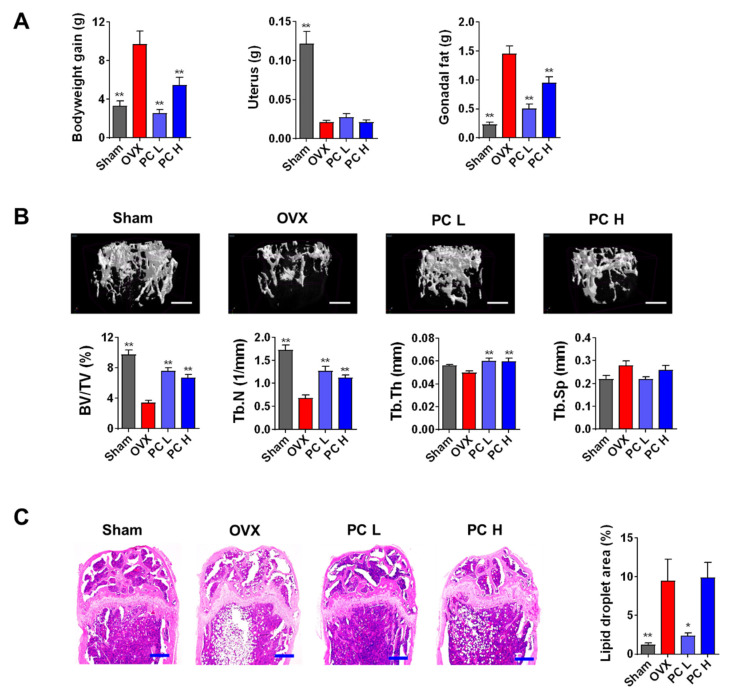
PC alleviates estrogen-deficiency- and high-fat-diet-induced bone loss and lipid accumulation in mice. (**A**) Body weight gain, uterine weight, and gonadal-fat weight during the experimental period. (**B**) Bone microarchitecture (scale bar, 0.5 mm) and trabecular bone parameters as shown by micro-computed tomography. (**C**) Marrow lipid droplets after hematoxylin and eosin staining (scale bar, 250 μm). Sham, sham-operated/vehicle; OVX, OVX/vehicle; PC L, OVX/low-dose PC treatment (10 mg/kg/day); PC H, OVX/high-dose PC treatment (20 mg/kg/day). BV/TV, bone volume to tissue volume ratio; Tb.N, trabecular number; Tb.Th, trabecular thickness; Tb.Sp, trabecular separation. Data are expressed as mean ± SEM (*n* = 8). * *p* < 0.05, ** *p* < 0.01 vs. OVX only.

We next focused on the expression of NFATc1 and its upstream transcription factor c-Fos to investigate the mechanism driving PC-mediated inhibition of osteoclast differentiation. NFATc1, a master transcription factor for osteoclastogenesis, modulates the expression of genes involved in osteoclastogenesis [20], while c-Fos induces NFATc1 expression [21]. PC-treated BMMs showed downregulated mRNA and/or protein expression of RANKL-induced NFATc1 and c-Fos (Figure 2B). Indeed, the expression of *Atp6v0d2*, cathepsin K, and *DC-STAMP*, which are late osteoclast-specific genes involved in bone resorption and cell-cell fusion [22], was downregulated in BMMs after treatment with PC (*p* < 0.01). Collectively, these results show that decreasing c-Fos protein levels by treatment with PC may lead to the suppression of NFATc1 expression.

### 3.2. PC Attenuates OVX-induced Bone Loss

The OVX mouse model is used for evaluating anti-osteoporotic effects of therapeutics and nutraceutics. This model mimics the symptoms of postmenopausal osteoporosis including hormonal-interruption-induced bone loss and lipid accumulation. Coupled with a high-fat diet (HFD), OVX aggravates bodyweight gain, and fat deposition in adipose tissues and the bone marrow. Lipid accumulation in the bone marrow is positively correlated with the risk of bone fracture in humans [23], while increased levels of marrow adipocytes worsen bone health and delay bone healing [24]. Our previous studies demonstrated that OVX mice fed an HFD showed markedly increased bone loss and lipid droplet levels [14,15]. Therefore, in this study, we evaluated the beneficial effects of PC (10 and 20 mg/kg/day) as a potent therapeutic against osteoporosis in OVX mice fed an HFD. Our results show that PC significantly inhibited bodyweight gain and increased gonadal fat levels (*p* < 0.01) (Figure 2A). The uterus weights of all the OVX-operated mice indicated atrophy, demonstrating successful induction of estrogen deficiency.

Morphological evaluation of bone microarchitecture, in particular of trabecular bone, can provide critical information on the degree of bone impairment and pathological state. µ-CT is a non-destructive quantitative assessment employed to define bone quality using calculated parameters such as bone thickness, separation, and density [25]. In general, the circumstantial evaluation of trabecular microstructure uses BV/TV, Tb.N, Tb.Th, and Tb.Sp. As shown in Figure 2B, mice administered different doses of PC showed thicker and more compact bone microarchitecture compared with those of mice not treated with PC. Morphometric analysis shows that treatment with PC significantly increased trabecular bone volume (BV/TV), number (Tb.N), and thickness (Tb.Th) compared with those of untreated mice (*p* < 0.01). In addition to alleviating bone loss, PC also decreased the number and areas of fat deposits in the bone marrow (*p* < 0.05) (Figure 2C). These findings imply that PC may have improved bone health and healing in vivo, suggesting that PC may be a promising candidate therapeutic for the management of osteoporosis in peri- and post-menopausal women. However, the mechanisms driving PC-mediated inhibition of lipid accumulation in the bone marrow need to be further studied.

### 3.3. Phytochemical Constituents of PC

**Figure 3:**
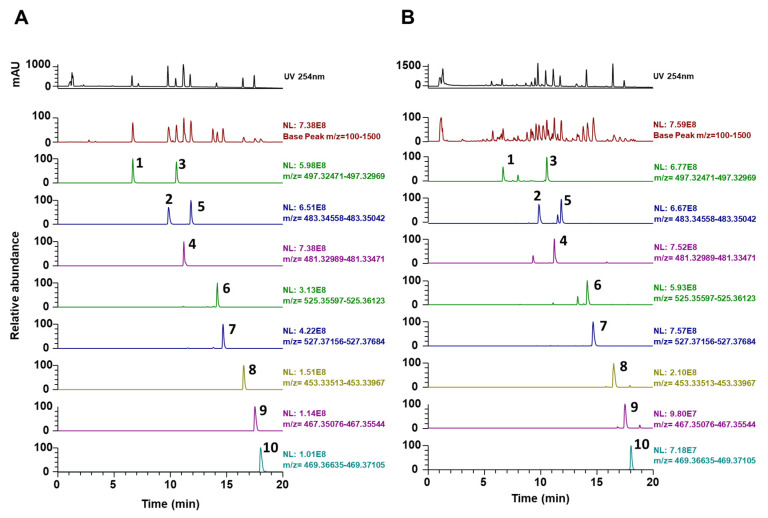
PC analysis using UHPLC-DAD–MS/MS. (**A**) Ultraviolet, base peak, and extracted-ion chromatograms of authentic reference standards. (**B**) Ultraviolet, base peak, and extracted-ion chromatograms of PC. 1, 6α-hydroxypolyporenic acid C; 2, dehydrotumulosic acid; 3, poricoic acid A; 4, polyporenic acid C; 5, 3-epidehydrotumulosic acid; 6, dehydropachymic acid; 7, pachymic acid; 8, dehydrotrametenolic acid; 9, dehydroeburicoic acid; 10, eburicoic acid.

Characterizing the ingredients of natural products can provide helpful information on their pharmacological and nutraceutical properties. *P. cocos* consists of two major (triterpenoids and polysaccharides) and minor (steroids, amino acids, choline, histidine, and potassium) groups of chemicals [10]. To determine the molecular basis for anti-osteoporotic effects of PC, we analyzed the chemical profile of PC using UHPLC–DAD–MS/MS. As shown in Figure 3 and Table 1, the PC used in our study contained 10 triterpenoids, including 6α-hydroxypolyporenic acid C, dehydrotumulosic acid, poricoic acid A, polyporenic acid C, 3-epidehydrotumulosic acid, dehydropachymic acid, pachymic acid, dehydrotrametenolic acid, dehydroeburicoic acid, and eburicoic acid, as determined using mass spectra and retention times.

All the constituents identified in our present study were in agreement with those identified in previous reports [9,16,26,27]. Polyporenic acid C inhibits M-CSF and RANKL-induced osteoclastogenesis in BMMs [28], while pachymic acid enhances odontoblast differentiation and dentin mineralization via induction of heme oxygenase-1 [29]. The other chemicals identified in this study have not, thus far, been associated with bone metabolism. Therefore, the beneficial effects of PC on estrogen-deprivation-induced bone loss may be due to the activity of the constituents mentioned above. In addition, numerous pharmacokinetic studies have reported that dehydrotumulosic acid, poricoic acid A, polyporenic acid C, dehydropachymic acid, pachymic acid, and dehydrotrametenolic acid are absorbed, distributed, metabolized, and excreted after oral administration of PC [9,26,30]. Further studies are needed to elucidate the precise mechanisms of components responsible for the anti-osteoporotic activity shown by PC and its effects on bone homeostasis.

## 4. Conclusions

To the best of our knowledge, this study is the first to demonstrate the possible beneficial effects of PC on bone health, as shown in a mouse model mimicking postmenopausal state. Our results show that PC suppressed osteoclastogenesis and osteolytic activity in our mouse model of OVX-induced estrogen deprivation. Moreover, oral intake of PC alleviated estrogen-deficiency-induced bone loss and lipid accumulation in the bone marrow. We also identified 10 phytochemicals with potential anti-osteoporotic activity. These findings indicate that PC is a potential natural therapy that can be used to manage postmenopausal osteoporosis. Further studies are warranted to access its effectiveness and safety in human.

## Figures and Tables

**Table 1:** Phytochemical constituents of PC as analyzed by UHPLC-MS/MS.

No	R_t_^*^ (min)	Calculated (m/z)	Estimated (m/z)	Adducts	Error (ppm)	Formula	MS/MS Fragments (m/z)	Identifications [References]
1	6.65	497.3272	497.3262	[M-H]^-^	−2.045	C_31_H_46_O_5_	419.2933, 405.2784, 403.2621	6α-Hydroxypolyporenic acid C [26]
2	9.83	483.348	483.3469	[M-H]^-^	−2.185	C_31_H_48_O_4_	437.3413, 423.3274, 405.3148, 389.2856	Dehydrotumulosic acid [27]
3	10.53	497.3272	497.3263	[M-H]^-^	−1.922	C_31_H_46_O_5_	423.2895, 379.2977, 211.1488	Poricoic acid A [16]
4	11.19	481.3323	481.3312	[M-H]^-^	−2.269	C_31_H_46_O_4_	435.3270, 421.3116, 311.2012, 97.0639	Polyporenic acid C [16]
5	11.83	483.348	483.347	[M-H]^-^	−2.058	C_31_H_48_O_4_	437.3439, 423.3260, 405.3159, 337.2531	3-Epidehydrotumulosic acid [27]
6	14.16	525.3586	525.3573	[M-H]^-^	−2.379	C_33_H_50_O_5_	465.3364, 355.2273,	Dehydropachymic acid [16]
7	14.67	527.3742	527.3732	[M-H]^-^	−1.955	C_33_H_52_O_5_	527.3356, 405.3150, 221.1897	Pachymic acid [16]
8	16.5	453.3374	453.3364	[M-H]^-^	−2.327	C_30_H_46_O_3_	453.3361, 435.3230, 371.2557, 337.2522	Dehydrotrametenolic acid [9]
9	17.53	467.3531	467.3519	[M-H]^-^	−2.442	C_31_H_48_O_3_	467.3520, 371.2567, 352.2839, 337.2527	Dehydroeburicoic acid [16]
10	18.04	469.3687	469.3677	[M-H]^-^	−2.095	C_31_H_50_O_3_	469.3675, 373.2722, 339.2684	Eburicoic acid [16]

^*^ R_t_, retention time.

## References

[1] Huang B., Wang J., Zhang X., Xie Z., Wu H., Liu J., Jie Z., Zhao X., Qin A., Fan S. (2019). Administration of SB239063 ameliorates ovariectomy-induced bone loss via suppressing osteoclastogenesis in mice. Front. Pharmacol..

[2] Riggs B.L. (2000). The mechanisms of estrogen regulation of bone resorption. J. Clin. Investig..

[3] Black D.M., Rosen C.J. (2016). Clinical practice. Postmenopausal osteoporosis. N. Engl. J. Med..

[4] An J., Yang H., Zhang Q., Liu C., Zhao J., Zhang L., Chen B. (2016). Natural products for treatment of osteoporosis: The effects and mechanisms on promoting osteoblast-mediated bone formation. Life Sci..

[5] Comhaire F.H., Depypere H.T. (2015). Hormones, herbal preparations and nutriceuticals for a better life after the menopause: Part I. Climacteric.

[6] De Franciscis P., Colacurci N., Riemma G., Conte A., Pittana E., Guida M., Schiattarella A. (2019). A nutraceutical approach to menopausal complaints. Medicina.

[7] Lee S., Choi E., Yang S.-M., Ryoo R., Moon E., Kim S.-H., Kim K.H. (2018). Bioactive compounds from sclerotia extract of *Poria cocos* that control adipocyte and osteoblast differentiation. Bioorg. Chem..

[8] Zhu L.-X., Xu J., Wu Y., Su L.-F., Ching Lam K.Y., Qi E.R., Dong X.-P., Chen H.-B., Liu Y.-D., Zhao Z.-Z. (2019). Comparative quality of the forms of decoction pieces evaluated by multidimensional chemical analysis and chemometrics: *Poria cocos*, a pilot study. J. Food Drug Anal..

[9] Qian Q., Zhou N., Qi P., Zhang Y., Mu X., Shi X., Wang Q. (2018). A UHPLC-QTOF-MS/MS method for the simultaneous determination of eight triterpene compounds from *Poria cocos* (Schw.) Wolf extract in rat plasma: Application to a comparative pharmacokinetic study. J. Chromatogr. B Analyt. Technol. Biomed. Life Sci..

[10] Ríos J.L. (2011). Chemical constituents and pharmacological properties of *Poria cocos*. Planta Med..

[11] Li X., He Y., Zeng P., Liu Y., Zhang M., Hao C., Wang H., Lv Z., Zhang L. (2019). Molecular basis for *Poria cocos* mushroom polysaccharide used as an antitumour drug in China. J. Cell. Mol. Med..

[12] Song D., Cao Z., Tickner J., Qiu H., Wang C., Chen K., Wang Z., Guo C., Dong S., Xu J. (2018). *Poria cocos* polysaccharide attenuates RANKL-induced osteoclastogenesis by suppressing NFATc1 activity and phosphorylation of ERK and STAT3. Arch. Biochem. Biophys..

[13] Ha H., Shim K.S., Kim T., An H., Lee C.J., Lee K.J., Ma J.Y. (2014). Water extract of *Acer tegmentosum* reduces bone destruction by inhibiting osteoclast differentiation and function. Molecules.

[14] Hwang Y.-H., Jang S.-A., Kim T., Ha H. (2019). Anti-osteoporotic and anti-adipogenic effects of *Rhus chinensis* nutgalls in ovariectomized mice fed with a high-fat diet. Planta Med..

[15] Hwang Y.-H., Jang S.-A., Kim T., Ha H. (2019). *Forsythia suspensa* protects against bone loss in ovariectomized mice. Nutrients.

[16] Jin J., Zhou R., Xie J., Ye H., Liang X., Zhong C., Shen B., Qin Y., Zhang S., Huang L. (2019). Insights into triterpene acids in fermented mycelia of edible fungus *Poria cocos* by a comparative study. Molecules.

[17] Asagiri M., Takayanagi H. (2007). The molecular understanding of osteoclast differentiation. Bone.

[18] Baron R., Neff L., Louvard D., Courtoy P.J. (1985). Cell-mediated extracellular acidification and bone resorption: Evidence for a low pH in resorbing lacunae and localization of a 100-kD lysosomal membrane protein at the osteoclast ruffled border. J. Cell Biol..

[19] Drake F.H., Dodds R.A., James I.E., Connor J.R., Debouck C., Richardson S., Lee-Rykaczewski E., Coleman L., Rieman D., Barthlow R. (1996). Cathepsin K, but not cathepsins B, L, or S, is abundantly expressed in human osteoclasts. J. Biol. Chem..

[20] Takayanagi H. (2007). The role of NFAT in osteoclast formation. Ann. N. Y. Acad. Sci..

[21] Matsuo K., Galson D.L., Zhao C., Peng L., Laplace C., Wang K.Z., Bachler M.A., Amano H., Aburatani H., Ishikawa H. (2004). Nuclear factor of activated T-cells (NFAT) rescues osteoclastogenesis in precursors lacking c-Fos. J. Biol. Chem..

[22] Soysa N.S., Alles N. (2016). Osteoclast function and bone-resorbing activity: An overview. Biochem. Biophys. Res. Commun..

[23] Fazeli P.K., Horowitz M.C., MacDougald O.A., Scheller E.L., Rodeheffer M.S., Rosen C.J., Klibanski A. (2013). Marrow fat and bone--new perspectives. J. Clin. Endocrinol. Metab..

[24] Ambrosi T.H., Scialdone A., Graja A., Gohlke S., Jank A.M., Bocian C., Woelk L., Fan H., Logan D.W., Schürmann A. (2017). Adipocyte accumulation in the bone marrow during obesity and aging impairs stem cell-based hematopoietic and bone regeneration. Cell Stem Cell.

[25] Marinozzi F., Marinozzi A., Bini F., Zuppante F., Pecci R., Bedini R. (2012). Variability of morphometric parameters of human trabecular tissue from coxo-arthritis and osteoporotic samples. Ann. Ist. Super. Sanita..

[26] Wu L.F., Wang K.F., Mao X., Liang W.Y., Chen W.J., Li S., Qi Q., Cui Y.-P., Zhang L.Z. (2016). Screening and analysis of the potential bioactive components of *Poria cocos* (schw.) wolf by HPLC and HPLC-MS(n) with the aid of chemometrics. Molecules.

[27] Feng G., Li S., Liu S., Song F., Pi Z., Liu Z. (2018). Targeted screening approach to systematically identify the absorbed effect substances of *Poria cocos* in vivo using ultrahigh performance liquid chromatography tandem mass spectrometry. J. Agric. Food Chem..

[28] Kwon J., Lee H., Yoon Y.D., Hwang B.Y., Guo Y., Kang J.S., Kim J.J., Lee D. (2016). Lanostane triterpenes isolated from *Antrodia heteromorpha* and their inhibitory effects on RANKL-induced osteoclastogenesis. J. Nat. Prod..

[29] Lee Y.H., Lee N.H., Bhattarai G., Kim G.E., Lee I.K., Yun B.S., Hwang P.H., Yi H.K. (2013). Anti-inflammatory effect of pachymic acid promotes odontoblastic differentiation via HO-1 in dental pulp cells. Oral Dis..

[30] Wang F.-Y., Lv W.-S., Han L. (2015). Determination and pharmacokinetic study of pachymic acid by LC-MS/MS. Biol. Pharm. Bull..

